# Invasive Group B Streptococcal Disease in the Elderly, Minnesota, USA, 2003–2007

**DOI:** 10.3201/eid1508.081381

**Published:** 2009-08

**Authors:** Neelay J. Kothari, Craig A. Morin, Anita Glennen, Delois Jackson, Jane Harper, Stephanie J. Schrag, Ruth Lynfield

**Affiliations:** Minnesota Department of Health, St. Paul, Minnesota, USA (N.J. Kothari, C.A. Morin, A. Glennen, J. Harper, R. Lynfield); University of Minnesota, Minneapolis, Minnesota, USA (N.J. Kothari); Centers for Disease Control and Prevention, Atlanta, Georgia, USA (D. Jackson, S.J. Schrag)

**Keywords:** Streptococcus agalactiae, streptococci, long-term care, antimicrobial resistance, elderly, microbial, Minnesota, USA, dispatch

## Abstract

In Minnesota, incidence of invasive group B streptococcal disease was 3 times greater in older adults in long-term care facilities than in older adults in community settings (67.7/100,000 vs. 21.4/100,000) during 2003–2007. The overall case-fatality rate was 6.8%, and concurrent conditions were common among both groups.

Invasive group B streptococcal (GBS) disease is a major cause of illness and death in older adults (*1*). A 2- to 4-fold increase in invasive GBS disease among US adults has been reported since the 1980s (*2*), and incidence increased 32% in adults from 1999 through 2005 (*1*). The objective of this study was to characterize the incidence and epidemiology of GBS disease in Minnesota among the elderly in long-term care facilities (LTCFs) and in the community.

## The Study

The Minnesota Department of Health conducts statewide, population-based surveillance for GBS disease as part of the Centers for Disease Control and Prevention Active Bacterial Core Surveillance Network/Emerging Infections Program. Invasive disease is defined as isolation of GBS bacteria from a normally sterile site, such as blood, pleural fluid, cerebrospinal fluid, joint fluid, or bone (*3*). To ensure completeness of reporting, the Minnesota Department of Health audits laboratories to identify all GBS bacteria–positive cultures from normally sterile sites. For each case, a standardized case report form is completed by hospital infection control practitioners. GBS isolates are sent to the Minnesota Department of Health Public Health Laboratory for susceptibility testing using broth microdilution. Erythromycin-resistant, clindamycin-susceptible isolates are tested for inducible clindamycin resistance by double-disk diffusion (D test). Interpretation is based on Clinical and Laboratory and Standard Institute protocols (*4*). Serotyping is performed at the Centers for Disease Control and Prevention by latex agglutination tests with rabbit antiserum to GBS capsular polysaccharide types Ia, Ib, and II–VIII (*5*). When latex tests are indeterminate, the Lancefield method is used (*6*).

The study comprised all Minnesota residents aged >65 years with invasive GBS disease during 2003–2007. LTCF residence was defined as living in an LTCF before the date of first positive culture. Resident addresses were checked by a reverse-address directory to determine whether they corresponded with the address of an LTCF. All other residents were defined as community dwelling. Incidence was calculated using 2000 census data. Analyses were conducted using SAS version 9.1 (SAS Institute, Cary, NC, USA); the χ^2^ test was used to evaluate differences in proportions for discrete variables.

A total of 723 cases of invasive GBS disease among persons >65 years of age were reported; 596 (82.4%) cases occurred among community residents, and 127 (17.6%) occurred among LTCF residents (Table 1). The overall incidence rate was 24.3 cases per 100,000 persons. Incidence did not vary significantly by year but did increase with age (19.3/100,000 at 65–74 years, 26.3/100,000 at 75–84 years, and 36.9/100,000 at >85 years; χ^2^ for trend = 44.4, p<0.001) and was higher among LTCF residents than among community residents (67.7/100,000 vs. 21.4/100,000; p<0.001). The overall case-fatality rate was 6.8 (8.7% LTCF vs. 6.4% community). Case-fatality rates increased as age increased (6.0% at 65–74 years, 6.8% at 75–84 years, and 8.2% at ≥85 years).

**Table 1:** Comparison of LTCF residents and community-dwelling elderly persons with invasive GBS disease, Minnesota, 2003–2007*

Characteristic	LTCF elderly, no. (%)	Community elderly, no. (%)
Total no. case-patients	127	596
Male gender	63 (49.6)	338 (56.7)
Type of infection†	63 (49.6)	297 (49.8)
Bacteremia without focus	24 (18.9)	55 (9.2)
Pneumonia	17 (13.4)	120 (20.1)
Cellulitis	5 (3.9)	47 (7.9)
Septic arthritis	5 (3.9)	24 (4.0)
Osteomyelitis	6 (4.7)	20 (3.4)
Abscess	1 (0.8)	3 (0.5)
Meningitis	13 (11.0)	58 (10.2)
Other or >2 types	7 (5.5)	27 (4.5)
Concurrent condition data collected	96 (75.6)	448 (75.2)
No concurrent conditions	5 (5.2)	52 (11.6)
1 concurrent condition	34 (35.4)	142 (31.7)
2 concurrent conditions	34 (35.4)	132 (29.5)
>3 concurrent conditions	23 (24.0)	122 (27.2)
Concurrent condition types‡	39 (40.6)	186 (41.5)
Diabetes	26 (27.1)	135 (30.1)
ASCVD	25 (26.0)	67 (15.0)
Congestive heart failure	13 (13.5)	23 (5.1)
Stroke	15 (15.6)	37 (8.3)
COPD	13 (13.5)	126 (28.1)
Cancer	44 (45.8)	195 (43.5)
Died	11 (8.7)	38 (6.4)

*LTCF residents were a median of 84 years of age; community residents, a median of 76 years of age (p<0.05). LTCF, long-term care facility; GBS, group B streptococcal; ASCVD, atherosclerotic cardiovascular disease; COPD, chronic obstructive pulmonary disease. †A single patient may have had >1 type of infection. ‡Percentages are of those with concurrent condition data collected.

The most common clinical presentation reported was bacteremia without focus (50.2%), followed by pneumonia (10.9%). LTCF residents (18.9%) were more likely than community residents (9.2%) to have pneumonia (p = 0.002) (Table 1). Blood (84.0%) was the most common site for isolation of GBS bacteria, followed by joint fluid (10.2%) and bone (3.3%). Other sites included peritoneal fluid (1.4%), pleural fluid (0.7%), and cerebrospinal fluid (0.4%).

Data on concurrent conditions were collected for 96 (75.6%) of 127 LTCF case-patients and 448 (75.2%) of 596 community case-patients. Of these, 176 (32.3%) had only 1 concurrent condition, 166 (30.5%) had 2 concurrent conditions, and 145 (26.6%) had >3 concurrent conditions. LTCF residents (94.8%) were more likely than community residents (88.4%) to have a documented concurrent condition (p = 0.06) (Table 1). Among case-patients with known concurrent condition status, 41% had diabetes mellitus and 30% had coronary artery disease; similar proportions were noted among LTCF and community case-patients. Congestive heart failure (26.0% vs. 15.0%, p *=* 0.009), stroke (13.5% vs. 5.1%, p = 0.003), and chronic obstructive pulmonary disease (15.6% vs. 8.3%, p = 0.026) were more common among LTCF residents. Cancer was more common among community residents (28.1% vs. 13.5%, p = 0.003) (Table 1). Cellulitis as a manifestation of invasive GBS disease was more likely in residents with diabetes than in those without diabetes (24.4% vs. 16.3%, p = 0.019).

GBS serotypes were obtained for 654 (90.5%) of 723 case-patients. Five serotypes, Ia (21.1%), Ib (11.0%) II (11.8%), III (11.3%), and V (35.0%), accounted for 94.6% of LTCF case-patients and 89.7% of community case-patients. Antimicrobial drug susceptibility data were obtained for 655 (90.6%) of 723 case-patients. All isolates were susceptible to penicillin. Susceptibility to erythromycin and clindamycin decreased during 2003–2007 (Table 2). Sixty percent of erythromycin-resistant, clindamycin-susceptible isolates had inducible clindamycin resistance as evidenced by a positive D test. During 2004–2005, 78% of erythromycin-resistant, clindamycin-susceptible isolates had inducible clindamycin resistance compared with 46% from 2006–2007 (p = 0.003). Serotype V was associated with higher rates of resistance than other serotypes to both erythromycin (46.7% vs. 27.9%, p<0.001) and clindamycin (28.4% vs. 12.9%, p<0.001). Serotype V was also associated with higher rates of inducible clindamycin resistance (88.6% vs. 43.4%, p<0.001).

**Table 2:** Susceptibility of invasive GBS disease to erythromycin and clindamycin, Minnesota, 2003–2007*

Susceptibility	No. (%) case-patients					p value†
	2003 (N = 133)	2004 (N = 127)	2005 (N = 131)	2006 (N = 143)	2007 (N = 121)	
Erythromycin susceptible	95 (71.4)	86 (67.7)	87 (66.4)	90 (62.9)	71 (58.7)	0.023
Clindamycin susceptible	114 (85.7)	105 (82.7)	108 (82.4)	116 (81.1)	92 (76.0)	0.057

*GBS, group B streptococcal. †χ^2^ test for trend.

## Conclusions

We found that rates of invasive GBS disease were substantial among the elderly and 3× higher among LTCF residents than elderly persons living in the community. These results are supported by an earlier report from Maryland that also found increased incidence of invasive GBS infections in LTCF residents (*7*). The reason for increased incidence in LTCF residents is not fully known. However, concurrent conditions, such as advanced age, diabetes, cirrhosis, and stroke, are known risk factors for GBS infection (*8*). In our study, concurrent conditions were common among both groups, however, concurrent condition types differed by group. Although higher rates of GBS disease among LTCF residents may be caused in part by differences in underlying concurrent conditions, other factors not collected as part of this study may also play a role. These factors include use of invasive devices (urinary catheters, intravenous catheters) and the possible role of person-to-person transmission of GBS bacteria in LTCF settings.

Case-fatality rates in this study were lower than those reported in other studies. National surveillance data (*1*) have shown a case-fatality rate of 13.1% for persons >65 years of age, and a similar study among LTCF residents showed a case-fatality rate of 16.7% (*7*).

Macrolide resistance is common, and increases in clindamycin resistance continue to occur among GBS strains. In the era of methicillin-resistant *Staphylococcus aureus* infections, nonpenicillin agents, such as clindamycin, are increasingly being used for empiric treatment of skin and soft tissue infections, but they may not provide adequate coverage if these infections are caused by GBS bacteria. Although β-lactams remain the preferred therapy for GBS infections, strains with elevated penicillin MICs have recently been reported (*9*–*11*).

The prevalence of serogroup V in this study is consistent with findings from other studies of adult populations (*12*,*13*) that show the recent emergence of this serotype. Serotype V has been associated with higher rates of antimicrobial drug resistance (*14*); thus, following trends in serotype prevalence may be useful. High rates of antimicrobial drug use in the elderly may result in further selection of serotype V, and resistance may increase in other serotypes. Molecular studies may be useful to further evaluate strains because serologically nontypeable strains contained specific capsular polysaccharide genes, including those for V (*15*). LTCF residents and persons with concurrent conditions should have high priority for vaccine administration after a vaccine becomes available. Vaccines should be multivalent; based on predominant serotypes, currently Ia, Ib, II, III, and V; and effective and immunogenic for older adults.
